# Anti-Adhesion Therapies in Inflammatory Bowel Disease—Molecular and Clinical Aspects

**DOI:** 10.3389/fimmu.2017.00891

**Published:** 2017-07-28

**Authors:** Sebastian Zundler, Emily Becker, Carl Weidinger, Britta Siegmund

**Affiliations:** ^1^Department of Medicine 1, University of Erlangen-Nuremberg, Translational Research Center, Erlangen, Germany; ^2^Department of Gastroenterology, Infectious Diseases and Rheumatology, Charité-University Medicine, Berlin, Germany; ^3^Berlin Institute of Health, Berlin, Germany

**Keywords:** inflammatory bowel diseases, ulcerative colitis, Crohn’s disease, vedolizumab, natalizumab, etrolizumab, gut homing, integrins

## Abstract

The number of biologicals for the therapy of immunologically mediated diseases is constantly growing. In contrast to other agents that were previously introduced in rheumatologic or dermatologic diseases and only later adopted for the treatment of inflammatory bowel diseases (IBDs), the field of IBD was ground breaking for the concept of anti-adhesion blockade. Anti-adhesion antibodies selectively target integrins controlling cell homing to the intestine, which leads to reduction of inflammatory infiltration to the gut in chronic intestinal inflammation. Currently, the anti-α4β7-antibody vedolizumab is successfully used for both Crohn’s disease and ulcerative colitis worldwide. In this mini-review, we will summarize the fundamental basis of intestinal T cell homing and explain the molecular groundwork underlying current and potential future anti-adhesion therapies. Finally, we will comment on noteworthy clinical aspects of anti-adhesion therapy and give an outlook to the future of anti-integrin antibodies and inhibitors.

## Introduction

Inflammatory bowel diseases (IBDs), such as Crohn’s disease (CD) and ulcerative colitis (UC), are characterized by chronically relapsing inflammation of the gut and are associated with considerable morbidity and reduced quality of life (1). The pathogenesis of IBD is still incompletely understood. However, environmental factors, genetic susceptibility, changes in the intestinal microbiome, and altered immune signaling in the gut have been identified to play an essential role during IBD development (2, 3). In particular, infiltration of various immune cells in the inflamed gut in IBD is a prominent feature of both CD and UC. These cells are targeted by most “traditional” IBD therapies including immunosuppressive agents and anti-tumor necrosis factor (TNF) antibodies. Yet, a significant portion of patients does not respond to such therapies, loses response or experiences side effects, underscoring the need for additional treatment concepts.

One such concept is anti-adhesion therapy. T lymphocytes are a crucial part of the intestinal immune (4, 5) system, and their numbers are mainly controlled by the balance of proliferation and apoptosis (6, 7) as well as by cell recruitment of circulating T cells from the bloodstream. The clinical use of antibodies like natalizumab or vedolizumab, which block surface molecules on T cells called integrins regulating their capacity to home to the gut, has conferred considerable attraction to intestinal T cell trafficking and the concept of anti-adhesion therapies. Meanwhile, several additional antibodies and compounds targeting distinct T cell trafficking steps are under development, and one or the other might soon contribute to a growing family of anti-trafficking drugs for the treatment of IBD.

In this mini-review, we will give an overview of the basic principles underlying intestinal T cell trafficking and summarize the translational relevance of these principles by highlighting the most important molecular and clinical aspects of current and future anti-adhesion therapies.

## Mechanisms of T Cell Trafficking

**Figure 1:**
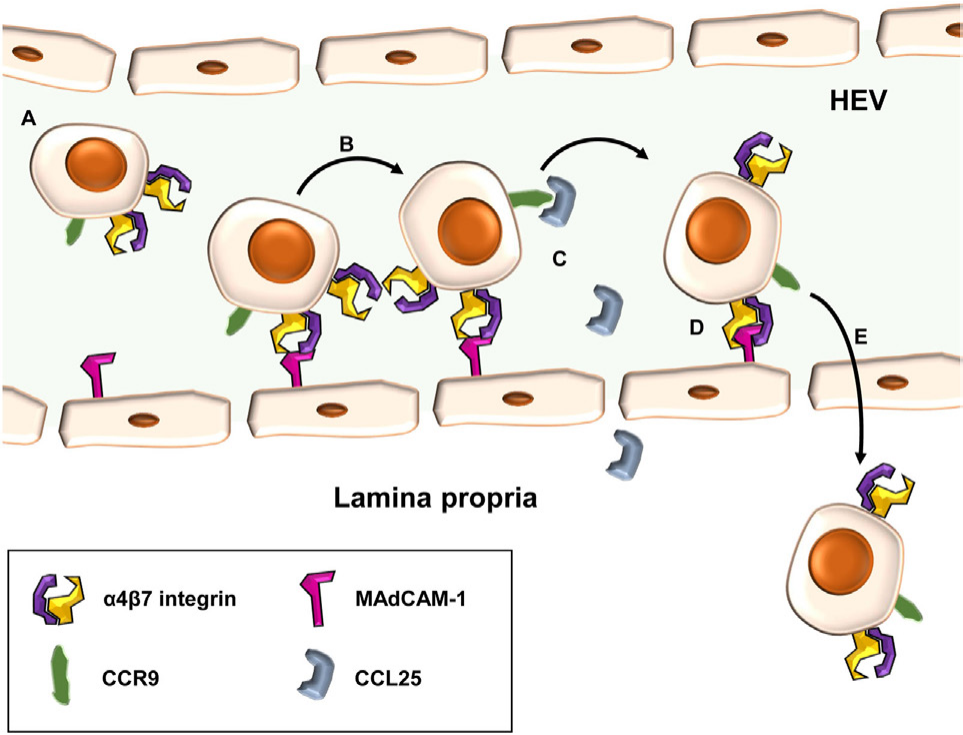
Principle of α4β7-mediated cell adhesion in the intestine. Gut-homing T cells carrying the α4β7 integrin and CC-chemokine receptor (CCR) 9 **(A)** may role along high endothelial venules (HEVs) of the gut by low-affinity interactions of α4β7 with mucosal vascular addressin cell adhesion molecule (MAdCAM)-1 **(B)**. Upon CCR signaling, e.g., *via* CC-chemokine ligand (CCL)-25 and CCR9 **(C)**, integrin-affinity modulation of α4β7 allows tight interaction with MAdCAM-1 and leads to firm adhesion of cells at the endothelial wall **(D)**. Subsequently, T cells may home para- or transcellularly to the lamina propria **(E)**.

A central event in the pathogenesis of T cell-dependent chronic intestinal inflammation is the homing of T lymphocytes to the gut (Figure 1). Homing describes a multistep process consisting of cell tethering to and rolling along activated endothelial cells, subsequent activation and firm adhesion of T cells, finally leading to their para- or transcellular transmigration from high endothelial venules (HEVs) into the tissue (8). To ensure that antigen-experienced T cells can reach their designated destination, a “zip code” like system of specific molecules controls homing to the intestinal lamina propria (LP). The expression of these molecules is primed during activation and expansion of naïve T cells after contact with their cognate antigen in the gut-associated lymphoid tissues. There, dendritic cells (DCs) (9), characterized by expression of CD103, not only present intestinal antigens to T cells and co-stimulate them, if applicable, but also produce retinoic acid (RA) through retinal aldehyde dehydrogenase. RA leads to upregulation of unique gut-homing markers including the integrin α4β7 and CC-chemokine receptor (CCR) 9 and, in turn, to loss of naïve T cell homing markers such as CCR7 (10–12).

After this switch in integrin expression, primed T cells can leave lymphoid organs to reenter the systemic circulation and adhere to intestinal HEVs expressing the addressin mucosal vascular addressin cell adhesion molecule (MAdCAM)-1 (13). MAdCAM-1 is the natural interaction partner of α4β7 integrin and, thus recognizing the “zip code” of gut-homing T cells (14). Unlike constitutively expressed selectins, integrins on T cells have to be activated in a process known as integrin-affinity modulation, before they can establish firm binding (15). This results in a conformation highly affinitive for the respective addressin. In contrast to other organs, where rolling is mainly mediated by selectins, weak and dynamic interactions of the low-affinity conformation of α4β7 with MAdCAM-1 are sufficient to induce tethering and rolling of T cells in the gut.

Rolling reduces the velocity of circulating T cells in the blood stream creating the basis for further homing and transmigration steps. Affinity modulation required for conformational change of α4β7 to its high-affinity state and subsequent firm adhesion is initiated by cell activation through chemokine receptor signaling. For instance, the CCL-25 secreted by LP cells in the small intestine, binds to CCR9, which is specifically expressed on gut-homing T cells. Subsequently, integrin heterodimers change from a folded position, in which the headpiece of the molecules is bent toward the plasma membrane and the addressin binding pocket is hidden, to an open conformation increasing not only the accessibility of the binding domain but also fully opening the pocket and enhancing its affinity (15, 16).

In addition to α4β7, other integrins like α4β1 may also contribute to adhesion of T cells to intestinal HEVs (17). Upon firm arrest of T cells, interactions of integrins with junctional adhesion molecules expressed on HEVs like JAM-1 contribute to para- or transcellular extravasation into the inflamed tissue (18).

Once homed to the gut, T cells contribute to immunological events depending on their designated role, such as T helper (Th) 1, Th2, Th9, Th17, cytotoxic T cells, or regulatory T cells (Tregs). However, trafficking of these cells is not necessarily finished, e.g., CCR7-dependent recirculation *via* lymphatic vessels (19) or sphingosine-1 phosphate-dependent exit to the blood stream has been described (20) and is reviewed elsewhere (21). Moreover, transforming growth factor β may trigger the upregulation of αEβ7 integrin, which cooperates with E-cadherin in the gut epithelial layer retaining T cells in or near the epithelium (22, 23).

It has been recognized more than two decades ago that all these mechanisms are not only academically interesting but also translationally relevant and allow targeted interference with the gut-homing process. Accordingly, targeted treatments for IBD interfering with the gut homing process have been developed and molecular and clinical aspects of these therapies will be discussed in the following paragraphs.

## Molecular Aspects of Anti-Adhesion Therapies

T cell trafficking includes a multitude of events such as priming, homing, recirculation, or retention, and all these steps are potential targets of therapy. So far, strategies impeding integrin-dependent cell adhesion to addressins have been most successful (24), and we will thus focus on these anti-adhesion therapies. Most importantly, the anti-α4-antibody natalizumab and the anti-α4β7-antibody vedolizumab reached clinical approval after large phase III studies (25–27).

**Figure 2:**
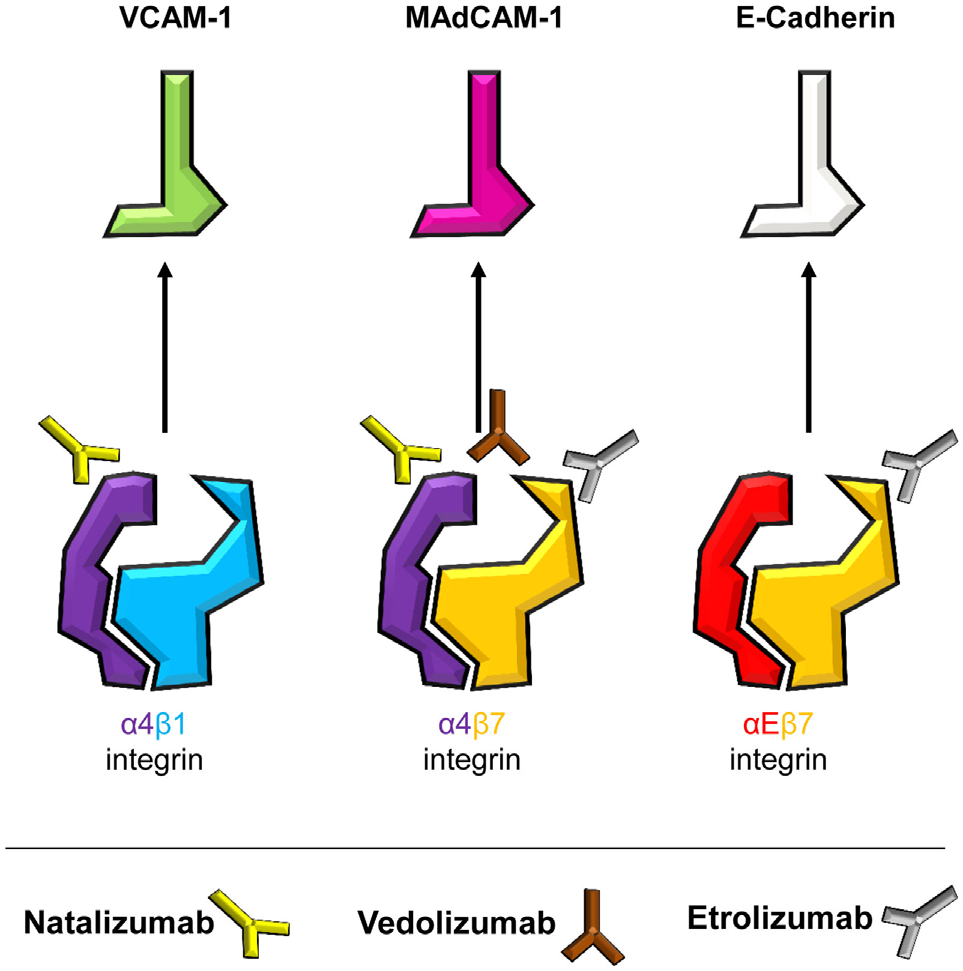
Specificities of current and potential future anti-adhesion antibodies. Integrins are heterodimers with an α- and a β-chain. Dimers containing α4 and β7 chains, i.e., α4β1, α4β7, and αEβ7 integrins, mediate intestinal T cell trafficking. By targeting monomers or heterodimers, different specificities of anti-integrin antibodies are achieved. Integrins and their respective ligands are indicated, and the antibodies natalizumab (anti-α4), vedolizumab (anti-α4β7), and etrolizumab (anti-β7) are depicted next to their respective target(s).

Yet, the divergent fate of these antibodies illustratively underscores redundancies and specificities (Figure 2) in integrin-dependent homing to the gut and other organs, based on the heterodimeric composition of integrins. An α and a β chain pair form an αβ heterodimer with most single chains combining with different other chains to form several distinct heterodimers. Thus, targeting integrins by antibodies on the monomer and heterodimer level results in coverage of a set of integrins or only one specific representative, respectively.

Both anti-α4 and anti-α4β7 strategies were initially evaluated in the cotton-top tamarine model of colitis, where they protected these animals from UC-like disease (28, 29), prior to testing of humanized antibodies in clinical trials. Soon after approval of natalizumab for CD, a report of progressive multifocal leukoencephalopathy (PML) was published (30), a severe infectious side effect deemed to arise from concurrent inhibition of α4β1-dependent homing *via* vascular cell adhesion molecule (VCAM)-1 to the central nervous system (31). Consistently, this has led to strong limitation or complete abandoning of natalizumab use in CD. On the other hand and matching with the current knowledge about T cell homing, vedolizumab, which is specific for the α4β7 heterodimer, has been successfully used for the treatment of both CD and UC for several years (32, 33) and has not been associated with infectious side effects in the central nervous system. The higher specificity of vedolizumab, however, also results in missing out alternative homing pathways as demonstrated by a study suggesting that homing *via* α4β1 might at least partially compensate for α4β7 blockade in CD patients treated with vedolizumab (17).

With the ongoing clinical studies of the anti-β7-antibody etrolizumab we are currently facing a new attempt to block α4β7 together with another integrin (34). Pan-β7 inhibition provides hopes that dual targeting of αEβ7 and α4β7 might increase therapeutic effects by additionally blocking intestinal retention of pathogenic T cells through E-cadherin (35). However, less gut specificity might be observed with β7 blockade since αEβ7 is also expressed by T cells in other tissues and might be important for the control of local infections there (36, 37). It will thus be an important task to determine potential infectious side effects of etrolizumab in the ongoing phase III trials. Moreover, it is not clear, whether anti-β7 antibodies impact CD103^+^ intestinal DCs (38). Since such DCs were proposed to be responsible for the induction of Tregs with anti-inflammatory properties under homeostatic conditions (39), it cannot be excluded that pan β7 inhibition reduces intestinal Treg cell numbers. However, it has also been shown that intestinal inflammation alters the role of these DCs switching their function to inducers of effector-like T cells (40), thus rather suggesting that anti-β7 treatment could help to reduce inflammation beyond the T cell level.

Taken together, the molecular mechanisms of targeting α4, α4β7, or β7 integrins in IBD show that it is not easy to find the optimum between the poles of maximally efficient gut homing blockade (i.e., inhibition of a plurality of responsible molecules) and selectivity (i.e., maximum safety). Therefore, further translational and empirical research is needed for elucidation of these challenging questions.

Such considerations get even more complicated when also taking the addressin side into account. Regarding the success of α4β7 inhibition, it seems logical that antibodies to MAdCAM-1 should result in similar clinical benefit. Yet, this black-and-white thinking does not match the myriads of grayscales in human biology since α4β7 is not only cooperating with MAdCAM-1 but also contains epitopes to bind to VCAM-1 and to fibronectin (41). This might be one explanation for the impression provided by early clinical studies that anti-MAdCAM-1 might not be as effective as vedolizumab (42, 43), although it has been claimed that vedolizumab does not interfere with α4β7 binding to VCAM-1 (41).

Another interesting molecular aspect of anti-adhesion therapies that is only beginning to be understood is the marked difference in the expression of integrins like α4β7 and αEβ7 on specific Th cell populations (35, 44). While Th2 and Th17 cells seem to express high levels of α4β7, Th1 and Th9 cells have low expression of α4β7. In contrast, αEβ7 is high in Th9 and Th17 but low in Th2 and Th1 cells (35). Since it is considered that CD is marked by Th1 and UC by Th2-like signaling (4, 45), differential expression of α4β7 might be one piece in the puzzle to explain, why the proportion of UC patients responding to treatment with vedolizumab seems to be higher compared with CD (25, 26). Moreover, it seems possible that assessment of individual or disease-specific Th cell profiles might help to optimize treatment by choosing antibodies most closely covering the respective subsets.

In conclusion, our understanding of the molecular mechanisms of gut homing has facilitated the development of novel therapies for IBD, but we are far away from a profound conceptual comprehension that includes an exact perception of the role of integrins and addressins in different tissues, with regard to different cellular subpopulations and concerning less prominent or rather overlooked “cross-interactions” between different homing pathways.

## Clinical Aspects of Anti-Adhesion Therapies

Blocking the migration of inflammatory cells into the target tissue is, as outlined earlier, an intriguing concept. The field was clinically implemented with the α4-antibody natalizumab. Clinical efficacy was proven first in a pilot study in CD (46), and subsequently in a phase III trial (47, 48). Here, patients with moderate to severe CD and an increase in C-reactive protein were randomized to receive 300 mg natalizumab or placebo at weeks 0, 4, and 8. Response by week 8, as indicated by a ≥70-point decrease from baseline in the CD activity index, sustained through week 12 in 48% of natalizumab-treated patients and in 32% of placebo-treated patients. This was statistically highly significant, and hence the primary endpoint of the study was met (48). These observations led to the approval of natalizumab for CD in North America. The enthusiasm for α4 blockade came to a sudden halt, when a fatal JC virus-related PML was reported upon natalizumab treatment (30), preventing the drug from approval in the European Union. The explanation for this side effect is rather obvious since anti-α4 equally hinders α4β1^+^ immune cells from not only infiltrating the gut but also the brain, hence impeding appropriate cerebral antiviral immunity.

Subsequently, the field moved on by developing more specific anti-adhesion strategies. The first and at this point only one with EMA approval for IBDs is the α4β7-antibody vedolizumab. Two large phase III trials led to approval (Table 1) (25, 26). Briefly summarized for UC, the primary endpoint at week 6, clinical response, showed significant differences (47.1% vedolizumab group vs. 25.5% placebo group) (25). Of the patients who responded to induction therapy at week 6, 88% were in remission after 104 weeks and 96% after 152 weeks of treatment (49). In CD, clinical remission showed a significant difference at week 6 (14.5% vedolizumab group vs. 6.8% placebo group) (26). Of all patients responding in week 6 who received vedolizumab continuously, 83 and 89% of patients were in remission after 104 and 152 weeks, respectively (50).

**Table 1:** Overview of clinical data from randomized-controlled studies on natalizumab, vedolizumab and etrolizumab in CD and UC.

	Efficacy		Important safety aspects
	CD	UC	
Natalizumab	Phase III: + 16% clinical response after 8 weeks vs. placebo (in patients with elevated CRP) (48)		Risk of PML (30)
Vedolizumab	Phase III: + 7.7% clinical remission after 6 weeks vs. placebo (26)	Phase III: + 21.6% clinical response after 6 weeks vs. placebo (25)	Nasopharyngitis, surgical site infection? (25, 26, 51)
Etrolizumab		Phase II: + 21% clinical remission after 10 weeks vs. placebo (34)	Influenza-like illness, arthralgia, and rash (34)

*Differences in the primary endpoint vs. placebo group are indicated, and most important side effects are noted. See text for details*.

*CD, Crohn’s disease; UC, ulcerative colitis; CRP, C-reactive protein; PML, progressive multifocal leukoencephalopathy*.

Besides these, initial phase III trials several real life registries from various countries have reported comparable efficacy (34, 49, 50, 52, 53).

In a German cohort with 212 consecutive patients with either CD or UC, clinical remission at week 14 was assessed (33). 23.7% of patients with CD and 23.5% with UC achieved clinical remission. One has to recognize that during the initial time period after approval mostly more refractory patients were exposed to vedolizumab. The cohort was then followed for 30 and 54 weeks, respectively, and included 67 CD and 60 UC patients. Primary endpoint was clinical remission at week 54, which was achieved in 21% of CD and 25% of UC patients, respectively (54).

It should also be mentioned that in comparison with anti-TNF antibodies, it seems that vedolizumab needs longer to manifest full effects (25, 26, 55). Regarding the abovementioned mechanistic aspects, it is tempting to speculate that this might be due to preserved function of T cells already present in the LP during the initial phase of vedolizumab treatment, while homing inhibition might only then lead to marked effects on T cell function, when a significant portion of these LP T cells undergoes apoptosis and replenishment is impeded.

Several other strategies are currently under clinical investigation including the anti-β7-antibody etrolizumab where a recent phase II trial for UC showed promising results and initiated a broad phase III study program (34). In a double-blind, placebo-controlled, randomized, phase II study including patients with moderately to severely active UC that did not respond to conventional treatment were randomized (1:1:1) to receive either etrolizumab 100 mg at week 0, 4, and 8 with placebo at week 2, 420 mg etrolizumab loading dose at week 0 followed by 300 mg at weeks 2, 4, and 8 or placebo. 124 patients were included and none of the placebo group patients reached the primary endpoint of clinical remission at week 10, whereas 21% of the etrolizumab 100 mg group and 12% in the 300 mg group met the endpoint. The authors conclude that etrolizumab showed clinical efficacy and hence α4β7 as well as αEβ7 might provide future therapeutic targets. Beside efficacy, the remarkable part of the study was that it provided for the first time a predictive biomarker for the responsiveness to an anti-inflammatory biological since αE expression in the intestinal mucosa correlated with a better response to etrolizumab treatment (34). In a follow-up study, these findings were specified and showed that high granzyme A and αE mRNA expression levels in colon biopsies revealed patients with UC more likely to respond to etrolizumab treatment (56).

Several other strategies target migration; one is approaching MAdCAM-1 on the endothelial site. A first dose-finding study indicated safety and efficacy in patients with UC (57). Very recently, the results of a phase II follow-up study were published. In this trial, patients were treated with subcutaneous injections of one of four doses (7.5, 22.5, 75, or 225 mg) of the anti-MAdCAM-1 antibody PF-00547659 or placebo. The primary endpoint was remission at week 12. This was met in three of the four verum groups (7.5, 22.5, or 75 mg), the highest difference in efficacy compared to placebo was observed in the 22.5 mg group (58).

## Safety

After the fatal complications observed under natalizumab treatment, none of the other strategies currently approved or studied revealed a new case of PML. A recent publication summarizes the collected safety data (May 2009–June 2013) from six studies of vedolizumab. Any patient that received ≥1 infusion of vedolizumab or placebo was included, and results were expressed as exposure-adjusted incidence rates with the number of patients experiencing the event per 100 person-years of exposure. The analysis included 2,830 patients with 4,811 person-years of exposure. Remarkably, no increased risk for any infection was associated with vedolizumab. Most important, up-to-date, no case of PML has been reported within this review or outside (59). The limitation of the study is the number of patients, while 2,789 had been exposed to ≥1 dose of vedolizumab, only 906 were exposed for ≥24 months and only 40 were exposed for ≥48 months (59).

Somewhat surprisingly, extra-intestinal symptoms in patients receiving vedolizumab are observed and are more common in those patients who respond to therapy (60). Recent data indicate that a shift in integrin expression under α4β7 neutralization toward a β1 upregulation results in an altered migrational behavior of immune cells in non-intestinal tissue including skin, joints, and lung (61, 62).

## Conclusion and Outlook

The discussed data indicate that anti-migrational strategies have found their way into clinical practice and the development of further anti-adhesion compounds together with other concepts like Janus kinase inhibitors, anti-IL-23p19 antibodies, or Smad7 blockade might provide optimized IBD treatment in the future. However, as outlined in the first paragraphs of this mini-review, a more detailed understanding of localized integrin expression is required to perform a more personalized treatment and identify the responding patients early on. However, first data indicate that this might become feasible.

## Author Contributions

SZ, EB, CW, and BS jointly wrote the manuscript and approved the final version.

## Conflict of Interest Statement

SZ has received research support from Takeda and Hofmann-La Roche. EB and CW have no competing interests. BS served as consultant for Abbvie, Falk, Janssen, Hospira, MSD, and Takeda; received speaker’s fees from Abbvie, Falk, Ferring, Hospira, Janssen MSD, and Takeda and a research grant from Pfizer.
